# Speed Bump Detection Using Accelerometric Features: A Genetic Algorithm Approach

**DOI:** 10.3390/s18020443

**Published:** 2018-02-03

**Authors:** Jose M. Celaya-Padilla, Carlos E. Galván-Tejada, F. E. López-Monteagudo, O. Alonso-González, Arturo Moreno-Báez, Antonio Martínez-Torteya, Jorge I. Galván-Tejada, Jose G. Arceo-Olague, Huizilopoztli Luna-García, Hamurabi Gamboa-Rosales

**Affiliations:** 1 Unidad Académica de Ingeniería Eléctrica, CONACyT—Universidad Autónoma de Zacatecas, Jardín Juárez 147, Centro Histórico, 98000 Zacatecas, Mexico; 2 Unidad Académica de Ingeniería Eléctrica, Universidad Autónoma de Zacatecas, Jardín Juárez 147, Centro Histórico, 98000 Zacatecas, Mexico; ericgalvan@uaz.edu.mx (C.E.G.-T.); eneldolm@uaz.edu.mx (F.E.L.-M.); morenob20@uaz.edu.mx (A.M.-B.) gatejo@uaz.edu.mx (J.I.G.-T.); arceojg@uaz.edu.mx (J.G.A.-O.); hlugar@uaz.edu.mx (H.L.-G.); hamurabigr@uaz.edu.mx (H.G.-R.); 3 Unidad Académica de Ingeniería I, Universidad Autónoma de Zacatecas, Jardín Juárez 147, Centro Histórico, 98000 Zacatecas, Mexico; omero.alonso@uaz.edu.mx; 4 Departamento de ingeniería, Universidad de Monterrey, Avenida Ignacio Morones Prieto 4500 Pte., Jesús M. Garza, 66238, San Pedro Garza García, Nuevo León, Mexico; antonio.martinez@udem.edu

**Keywords:** smart car, surface monitoring, speed bump detection

## Abstract

Among the current challenges of the Smart City, traffic management and maintenance are of utmost importance. Road surface monitoring is currently performed by humans, but the road surface condition is one of the main indicators of road quality, and it may drastically affect fuel consumption and the safety of both drivers and pedestrians. Abnormalities in the road, such as manholes and potholes, can cause accidents when not identified by the drivers. Furthermore, human-induced abnormalities, such as speed bumps, could also cause accidents. In addition, while said obstacles ought to be signalized according to specific road regulation, they are not always correctly labeled. Therefore, we developed a novel method for the detection of road abnormalities (i.e., speed bumps). This method makes use of a gyro, an accelerometer, and a GPS sensor mounted in a car. After having the vehicle cruise through several streets, data is retrieved from the sensors. Then, using a cross-validation strategy, a genetic algorithm is used to find a logistic model that accurately detects road abnormalities. The proposed model had an accuracy of 0.9714 in a blind evaluation, with a false positive rate smaller than 0.018, and an area under the receiver operating characteristic curve of 0.9784. This methodology has the potential to detect speed bumps in quasi real-time conditions, and can be used to construct a real-time surface monitoring system.

## 1. Introduction

The Internet of Things (IoT) is a recent communication paradigm in which objects of everyday life are envisioned to be able to transmit and receive digital information to generate machine-machine and human-machine interactions. This concept would enable an easier access and interaction with all sorts of objects, from home appliances, to monitoring devices, smart phones, wearables, vehicles, and so on. Such devices would generate a huge amount of data that may enable the development of advances in home and industrial automation [1].

The application of IoT to an urban context, the “Smart City”, is of particular interest, since it has a direct relation with sustainability and to the rise of new Internet technologies [2,3]. No matter the type of implementation, Smart Cities aim at making a better use of public resources, increasing the quality of the services offered to the citizens [1]. The development of new IoT technologies may bring a number of benefits in the management and optimization of traditional public services, such as public transportation, street lighting, surveillance and maintenance of public areas, new services upon those provided by the IoT [4].

Among the current challenges of the Smart City, traffic management is one of the most difficult. Traffic monitoring systems that obtain information via cameras installed throughout a city have already been deployed [1,5], and the GPS systems of modern vehicles are also being evaluated as a better source of information [6,7,8]. This data is meant to be used by authorities in order to predict and avoid road congestions. Nevertheless, there is still valuable information that is not being gathered by machines, but rather by humans, such as road surface monitoring, currently performed by road workers.

Road surface condition is one of the main indicators of road quality, and it may drastically affect fuel consumption and the safety of drivers and pedestrians. Abnormalities in the road such as manholes and potholes can cause accidents when not identified by the drivers. Furthermore, human-induced abnormalities, such as speed bumps, could also cause accidents. While said obstacles ought to be signalized according to specific road regulation, they are not always correctly labeled.

The development of a machine-driven system for the monitoring of road surface conditions is challenging, but it would largely benefit society. For instance, information gathered via rich sensing could be used to annotate maps, thereby allowing travel route optimization based on, among other variables, the amount of speed bumps. Doing so would minimize fuel consumption due to the constant acceleration and deceleration, therefore, also the reducing the environmental impact, and would also avoid the premature wear of the vehicles. Some researchers have already worked on monitoring surface condition of the road, Devapriya et al. [9] proposed a real-time speed bump detection by analyzing the images from the road and applying computer vision enhancement based on a Gaussian filter and a connected component approach. The system was able to detect the speed bumps with a true positive rate between 30% and 92%. Nevertheless, in order to achieve the higher rate, speed bumps had to be correctly painted and labeled. Eriksson et al. [10] developed the patrol system, which uses the 3-axis accelerometer and GPS mounted on the dashboard of the vehicle to monitor the road surface and detect potholes. This system collects data, then a series of signal processing filters are used to reduce the noise, and finally a machine learning algorithm performs a classification to classify potholes with a false positive rate of less than 0.2% in controlled conditions.

Similarly, Chen et al. [11] developed a system that also collects information from the 3-axis accelerometer and GPS of the vehicle. This system analyses the power spectral density (PSD) to detect pavement roughness using Fourier transformations. The international roughness index is calculated based upon PSD, but this system does not provide the proper location of the speed bumps. Mohan et al. [12] developed a system called Nericell that uses the accelerometer, microphone, GSM radio, and GPS sensors present in smart phones to monitor road and traffic conditions. It was used to detect potholes, braking, speed bumps, and honks. The system was tested using a dataset of 62 low-speed bumps and 39 high-speed bumps yielding a false negative rate of 37% and 41%, respectively. Recently, Arroyo et al. [13] presented a new methodology to identify sudden driving events (i.e., acceleration, steering, braking) and road bumps from the inertial and GPS sensors using a smart phone and an Adaptive Fuzzy Classifier, the authors proposed an on-line calibration method to adjust the decision thresholds of the Membership Functions to the specific vehicle dynamics, the methodology obtained a precision performance of 0.87 and a 0.91 recall. Similar to said approach Aljaafreh et al. [14] proposed a speed bump detection method based on a fuzzy inference system, the fuzzy inference system detected and recognized the speed bumps from the variance of the vertical acceleration and the speed of the vehicle using the embedded accelerometer in a smart phone, the proposed method was tested and evaluated under different speed levels, the authors reported a promising for bumps detection system, nevertheless, actual performance of the test is missing. De Silva et al. [15] explored a system based on filters named BusNet to locate potholes along the path traversed by public transportation buses, the author reported an accuracy performance of 70 to 80%. Astarita et al. [16], developed a multi smart phone system to detect speed bumps, using the accelerometer the system was trained to detect potholes and bumps, the algorithm developed to detect road bumps and potholes was based on the analysis of the acceleration signal in terms of high-energy events; three filters were applied on the original signal, the results obtained a detection up to 90% of bump events whereas the rate of false positive events was about 35%. Later, González et al. [17] developed a methodology to analyze the road surface condition using a smart-phone and the embedded sensors, the approach used the the accelerometer data and a Bag of Words representation in order to characterize the road surface condition, the authors benchmarked six different algorithms for the classification, for the detection of speed bumps the authors reported an cross-validation AUC ranging from 0.82 up to 0.944. Nature driven approaches such a genetic algorithms were also explored [18], Salari and Yu [19] explored the use of genetic algorithms using images as a source of information to detect potholes and pavement distress, the proposed methodology was able to detect the pavement distress with an accuracy of 97%.

In this research we propose a novel methodology to detect speed bumps in quasi real-time conditions. Our approach is based on a multivariate genetic algorithm fed with information from an accelerometer, a GPS, and a gyro sensor connected to an IoT device.

## 2. Materials and Methods

**Figure 1:**
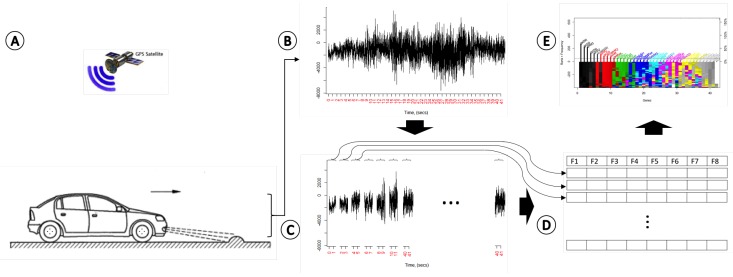
Flow chart of the proposed methodology. Several sensors connected to an IoT device (**A,B**); The real-time information of the sensors is trimmed intoltwo-second windows of data (**C**); Several statistical features that characterize the whole data are extracted from each data window (**D**); A machine learning approach based on genetic algorithms is applied to find a logistic model that can be used to accurately detect speed bumps (**E**).

Figure 1 shows the methodology overview. Briefly, in order to detect speed bumps, we first gather information from several sensors connected to an IoT device Figure 1A,B, specifically a Raspberry Pi. The real-time information of the sensors is trimmed into two-second windows of data Figure 1C. Then, several statistical features that characterize the whole data are extracted from each data window Figure 1D. Finally, a machine learning approach based on genetic algorithms is applied to find a logistic model that can be used to accurately detect speed bumps Figure 1E. Each stage is explained in detail next.

### 2.1. Data Collection

This section details the hardware and software setup used in this work. Besides the trimming of the data into two-second samples, data was not preprocessed.

#### 2.1.1. Hardware Setup

We aimed at implementing the data collection on low-cost hardware using an open software approach, therefore, after extensive research we determined to use a Raspberry Pi 3 development board. In order to characterize the movement of the car three sensors were used: a GPS, an accelerometer, and a gyro sensor. Table 1 shows the specifications of each sensor and the board.

**Figure 2:**
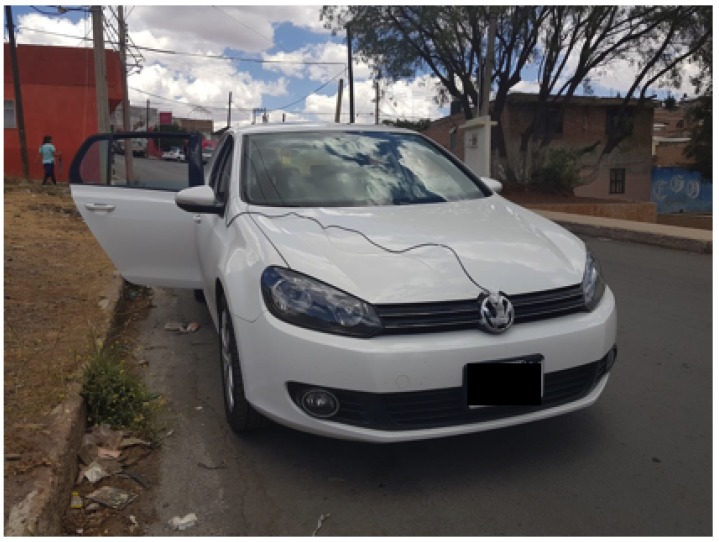
Field experiment set up.

The accelerometer and the gyro sensor were mounted to a test vehicle in the middle of the front bumper, as shown in Figure 2, and connected using a shielded UTP cable to the GPIO pins of the Raspberry Pi. Both sensors were set to a sample rate of 95 Hz. The GPS sensor was mounted on top of the vehicle, and its receiver was set to a 1 Hz refresh rate. Additionally, a physical trigger (i.e., a button) was attached to the GPIO of the Raspberry Pi to label the true position of speed bumps. The labeling was performed by a person sitting in the passenger seat during data collection.

A Python program ran on the board while the vehicle was in motion. It collected the following data: time, latitude, longitude, elevation, speed, gyro *x*-, *y*-, and *z*-axis, accelerometer *x*-, *y*-, and *z*-axis, and trigger. Only the information retrieved from the accelerometer, gyro, and trigger were used to detect speed bumps. The time, speed, and GPS information were used to locate the speed bumps in a map.

#### 2.1.2. Location

This research was conducted in Zacatecas, Mexico. This city is located in the north of Mexico, has an average elevation of 2,460 m. above sea level, and a mountain topology [20]. Several streets in low-traffic areas with a high-rate of speed bumps were chosen to perform the experiments. The test car was set to 20 km/h in order to simulate a residential-zone driving style. A total of ten different laps were performed, yielding 14,090 data samples.

### 2.2. Feature Extraction

Most of current approaches of speed bump detection rely only on simple thresholds or a combination of digital filters to suppress noise and a classification model. Nevertheless, since we dealt with a large amount of data, our approach was based on machine learning algorithms [21,22]. In order to make sense of the data, and based on previous efforts [23], seven features were extracted from each data window, all detailed in Table 2. This reduced the 1140 time-dependent samples (6 sensors at 95 Hz) from each two-second window to 42 time-independent features. The final dataset contained 752 observations and 42 features plus the ground true speed bump label.

### 2.3. Model Construction

Nature-driven approaches are currently getting attention for their lower computational requirements to solve complex problems [24]. Among those, one of the most used are genetic- or evolution-driven approaches. These methodologies try to mimic the evolutionary process, generating models composed of different features that reproduce, mutate, migrate, etc., and where the fittest models prevail.

For this research, we used GALGO [25], a powerful multivariate feature selection strategy based on genetic algorithms. With it, and from the dataset containing the two classes (i.e., speed bump and no speed bump), the genetic algorithm evolved a set of random multivariate models into highly accurate models. Features appearing multiple times in these models suggested an importance for the classification problem. Therefore, the frequency with which a feature appeared in these models was computed and used to rank features. Then, based on this rank, a forward selection and backwards elimination strategy was used to select a representative model.

The dataset was split into train (80%) and test (20%) samples, and the genetic search was carried out only taking the train samples into consideration. One thousand 5-feature models evolved throughout 200 generations, where fitness was evaluated as the accuracy of the model following a 3-fold cross-validation strategy (70% train and 30% test) within the previously defined train samples.

A logistic regression function was used as the classification method in the genetic search, as defined in:

(1) $$F\left(x\right)=\frac{1}{1+e^{-z}}$$

where F(x) is the probability of a sample being a speed bump. Here, *z* is a linear combination of features of the form $\beta_{0}+\beta_{1}x_{1}+\beta_{2}x_{2}+\ldots +\beta_{5}x_{5}$.

After the genetic search was completed the forward selection and backwards elimination process was performed using the whole train subset. The forward selection algorithm created models by adding one feature at a time, from the highest- to the lowest-ranked, and evaluated the performance of each model. The features from the model that achieved the highest accuracy from this subset of models were kept, and the rest were disregarded. Then, the backward elimination was performed to avoid redundant information and further reduce the amount of features to be used. This process consisted in evaluating the forward selection model after one of its features had been removed. The evaluation was performed for the removal of each feature. Had the removal of a feature not decreased the accuracy of the model, that feature was definitely removed from the model. This process was repeated until all features impacted the accuracy of the model, this process, yielded a best performing logistic model, said model was used for the speed bump detection. Accuracy was measured throughout this whole process using the same methodology as in the genetic search. Finally, in order to measure the true performance of the model, it was evaluated using the test subset.

## 3. Results

**Figure 3:**
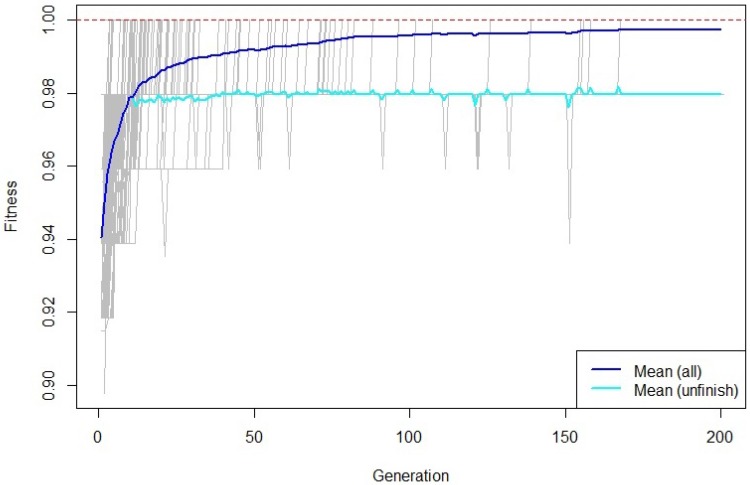
Evolution of the accuracy.

**Figure 4:**
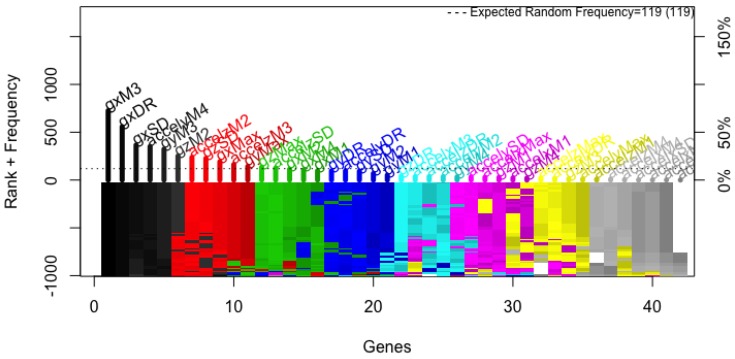
Rank stability in 1000 models. The y-positive axis shows the number of times each feature was included in a given model, the frequency ranking. The *y*-negative axis shows the color coded rank of each feature as each model was generated, for example, the sixth most frequent feature when all models had been generated (dark gray) was ranked lower (dark red) when only half of the models had been evolved. The *x*-axis shows the features ordered by rank. Starting color for each feature is assigned accordingly to the feature descending rank (from black down to white).

Data collection resulted in a total of 752 two-second recordings, with 42 features and a ground true label extracted from each, yielding a 752 × 43 matrix. Using this dataset, the genetic search generated 1000 models. Figure 3 shows the average accuracy throughout the 200 generations in which models evolved, and highlights that accuracy converged. That is, no more generations were needed. Similarly, Figure 4 shows that the frequency in which features appeared in the models had stabilized. There, it can be seen that the eleven most frequent features are above the expected random frequency, thus, even with more models the ranking would have stayed the same.

**Figure 5:**
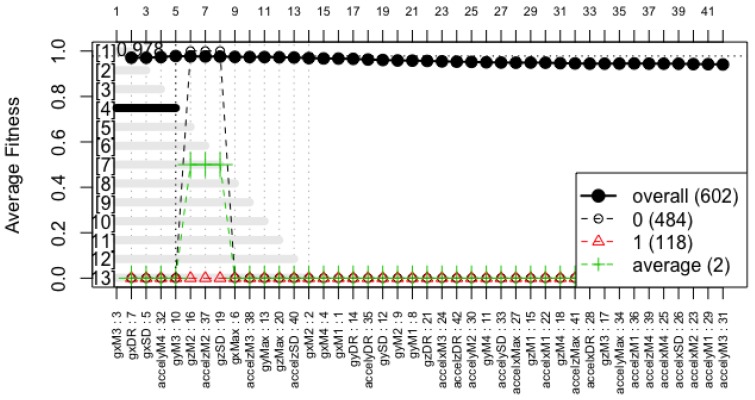
Forward selection model.

The forward selection model yielded a 5-feature model, as shown in Figure 5, and the backward elimination strategy removed one feature from it without losing accuracy, resulting in a representative model constructed with only 4 features. The specific features used to construct this model, their coefficients within the linear equation, and their *z*-value and corresponding *p*-values are detailed in Table 3.

**Figure 6:**
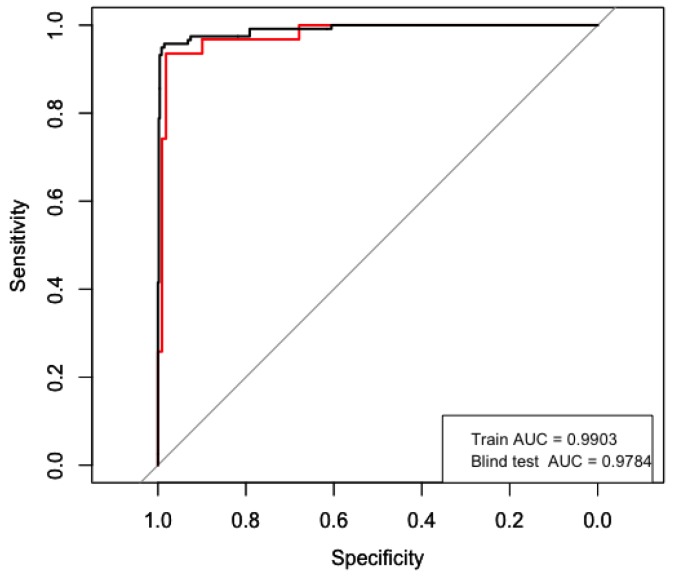
ROC curve of the representative model, black line = train performance, red line = blind performance.

The final model was validated in the test dataset. To measure its performance, a receiver operator characteristic (ROC) curve was computed and the area under this curve (AUC) was measured. Figure 6 shows the ROC curve, which had an AUC of 0.9784. The model also achieved an accuracy of 0.9714 (95% confidence interval = [0.9285,0.9922]) a false positive rate of 1.42%, and a true positive rate of 93.5%.

**Figure 7:**
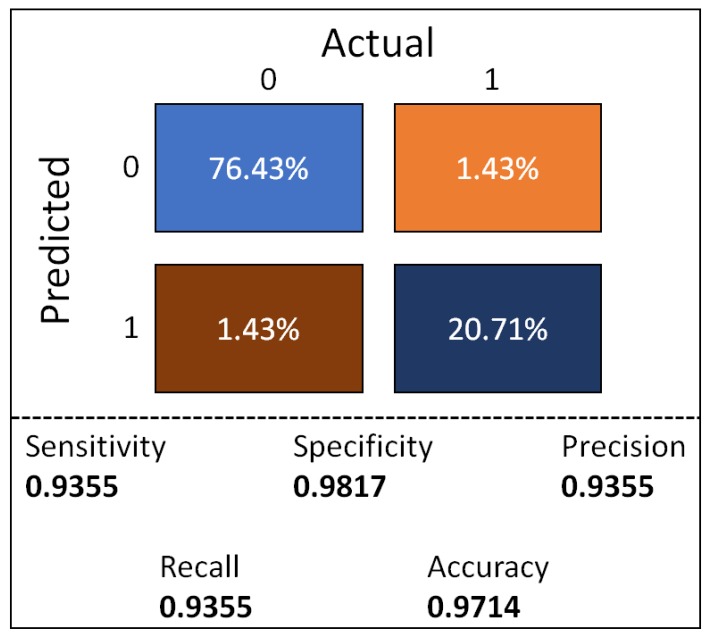
Confusion matrix performance in the blind data set, light blue = true negative, orange = false negative, brown = false positive, dark blue = true positive.

In Figure 7, the confusion matrix for the final model tested in the blind data set is shown, the final model achieved a precision/recall of 0.9355.

## 4. Discussion

In this work, we collected raw data from an accelerometer and a gyro sensor and analyzed it as two-second windows of data in order to characterize the information using statistical features that described the behavior of each feature. The statistical features were fed into a genetic-based algorithm that evolved one thousand random 5-feature models throughout 200 generations. Based on this results, a forward selection and backwards elimination process yielded an accurate 4-feature model, as the blind evaluation showed.

The proposed model included the following features: Skewness and dynamic range of the gyro’s *x*-axis information, skewness of the gyro’s *y*-axis information, and kurtosis of the accelerometer’s *y*-axis information. Table 3 shows that all features had a significant *p*-value, suggesting that all of them contribute to the performance of the model. While many approaches use only maximum values of the measured signals in order to detect speed bumps, we can see that non of these features were included in the proposed model. This may indicate that such a measure is no the best way to characterize the information being gathered, and that other characteristics, such as the kurtosis and dynamic range (which depends partially on the maximum value of the signal) may be better sued for the task.

In addition, the model hereby proposed was extremely accurate no only when evaluated inside the training dataset (results not shown), but also within a blind evaluation setting, in which the dataset to be classified had never before been taken into account, and was thus not accounted in the calibration of the coefficients. This performance suggests that the model is robust, and that it could be applied in new scenarios without the need of a recalibration.

Table 4 shows a full comparison between the results obtained with the proposed methodology and those yielded by similar approaches. As it can be seen, our approach clearly outperforms the rest. We can see that our approach had less false positives than the approach presented by Pothole Patrol [10]. When comparing our results against Wolverine [26] we can see that our approach did obtained a 10 fold lower false negative ratio, on the other hand when comparing accuracy we also obtained a higher accuracy of 0.9714, nevertheless, the authors acknowledge that accuracy may not be a good performance measurement for unbalance dataset, despite such drawback, in Figure 7 we can see the confusion matrix for the model on the blind dataset, the obtained performance exhibit a high sensitivity and specificity of 0.9355 and 0.9817 respectively, the model obtained a precision of 0.9355, such performance makes the model suitable for a high detection rate with a very low error rate.

In order to test the bias towards a specific data set partition, the whole experiment was executed 5 times with different 80% (train/test)–20% (blind-test) distributions. Table 5 describes the full performance of the proposed methodology, where it can be seen that the performance is consistent along the different data set partitions. This indicates that the proposed methodology is robust and stable despite the data partition. Since the range of values collected from each sensor may greatly vary, we carried out a test to establish any potential bias towards a specific range of sensor values. For this, a z-normalization of the data was carried out, and the whole experiment was executed 5 times with different 80% (train/test)–20% (blind-test) distributions. Table 6 describes the full performance of the proposed methodology using the z-normalized data, where it can be seen that the performance is consistent along the different data set partitions and the average AUC (0.981) was almost identical to the one obtained with raw data (0.987). Such a small difference indicates that the proposed methodology is robust and stable despite the value range of the sensors and data partition scheme. Furthermore, it indicate that the obtained logistic model can be transferred to an IoT device using either the z-normalized or raw data depending on its computing capabilities.

One of the strongest points presented in this research is the use of a multivariate model for speed bump detection targeting quasi-real time applications. The presented methodology builds a model using a logistic regression as a fitness function; the procedure is consistent and adapts to sparsity. Once the model is trained and refined, the model could be transferred to a mobile application, and said application would only be using the logistic model and the values of the coefficients optimized in the training stage, thus, it would not need to be retrained once in the mobile device, avoiding computation complexity.

## 5. Conclusions

The proposed methodology was able to collect and process the raw data of the accelerometer and gyro sensors, then a multivariate model was created by means of a genetic algorithm. The final model included only four features and yielded an AUC of 0.9784 and an accuracy of 0.9714 on a blind test, with a false positive rate smaller than 0.018%. The model successfully detected the speed bumps even on the blind unseen dataset.

The logistic model had features that were not previously studied by other authors and had the potential to detect in a quasi-real time scenario the speed bumps. This information could be used to develop a real-time road surface monitoring system. The system can also be used to construct real-time smart routes in order to avoid obstacles, thus, reducing the fuel consumption and the environmental impact of the cars being driven.

## 6. Future Work

We believe that this systems could be further simplified by using only the information from one source, either the accelerometer or the gyro. In this work, the removal of one source of information yielded results not as good as the ones being presented. However, a better characterization of the information, that is, extracting more statistical features from each two-second windows, could lead to an enhanced performance, even when only taking into account one source of information. Furthermore, the forward selection and backward elimination strategies could also be redesigned to obtain a more accurate model, even when working with only one source of information. A deep learning and big data-oriented algorithms will be investigated in order to assist large scale deployment and complex scenarios. In addition, were the speed bump location system to be included in government vehicles, such as the ones used by the transit department, we could map the surface road quality of the whole city of Zacatecas and have continuous updates.

## Figures and Tables

**Table 1:** Hardware specifications.

Hardware	Description
Raspberry Pi 3	Low cost ARM computer with a Quad Core 1.2GHz 64-bit CPU, 1 GB RAM, wireless LAN and Bluetooth, GPIO, and 4 USB 2.0 ports, power consumption: 800 mA
MPU6050	This sensor includes a MEMS-accelerometer and a MEMS-gyro in a single chip. It includes 16-bit analog to digital conversion capabilities for each channel, capturing the *x*-, *y*-, and *z*-channel at the same time, power consumption: 3.9 mA
7″ multi-touch screen	An 800 × 480 display that connects via the DSI port of the Raspberry Pi. It supports up to 10-finger touch, power consumption: 600 mA
BU-353-S4	A SiRF Star IV powered GPS sensor with a 1 Hz. refresh rate, and a < 2.5 m. accuracy, power consumption: 55 mA.
TL-PB10400	10400 mAh external battery

**Table 2:** Extracted features.

Feature	Formula
Mean (M1)	$\bar{x}=\frac{1}{n}\sum_{i=1}^{n}X_{i}$
Variance (M2)	$\sigma^{2}=\frac{\sum_{i=1}^{n}{\left(X_{i}-\bar{X}\right)}^{2}}{N}$
Skewness (M3)	$\gamma_{1}=\frac{\frac{1}{n}\sum_{i=1}^{n}{\left(x_{i}-\bar{x}\right)}^{3}}{{\left[\frac{1}{n-1}\sum_{i=1}^{n}{\left(x_{i}-\bar{x}\right)}^{2}\right]}^{3/2}}\;,$
kurtosis (M4)	$K=\frac{\sum_{i=1}^{N}{\left(X_{i}-\bar{X}\right)}^{4}}{Ns^{4}}-3$
Standard Deviation	$\sigma =\sqrt{\sigma^{2}}$
Max	$X_{\left(n\right)}=\max\left\{\;X_{1},\ldots ,X_{n}\;\right\}.$
Dynamic range	$DR=X_{\left(n\right)}-\min\left\{\;X_{1},\ldots ,X_{n}\;\right\}$

$X_{i}$ is the *i*th raw signal value within the two-second window being processed.

**Table 3:** Final model.

Feature	Coefficient	Std. Error	*z*-Value	*p*-Value
Intercept	−8.066	1.254	−6.433	1.250 × 10${}^{-10}$
gxM3	−1.131	4.370 × 10${}^{-1}$	−2.589	9.633 × 10${}^{-3}$
gxDR	5.070 × 10${}^{-4}$	5.974 × 10${}^{-5}$	8.487	< 2 × 10${}^{-16}$
gyM3	2.500	7.024 × 10${}^{-1}$	3.560	3.710 × 10${}^{-4}$
ayM4	−7.382 × 10${}^{-1}$	2.335 × 10${}^{-1}$	−3.162	1.569 × 10${}^{-3}$

gxM3 and gxDR are the skewness and dynamic range of the gyro sensor’s *x*-axis information, respectively; gyMR is the skewness of the gyro sensor’s *y*-axis information; and ayM4 is the kurtosis of the accelerometer’s *y*-axis information.

**Table 4:** Comparison of similar approaches.

Author	Approach	Performance
Devapriya et al. [9]	Computer vision	30–92% TPR
Eriksson et al. [10]	Accelerometer and GPS	0.2% FPR
Mohan et al. [12]	Accelerometer, microphone, GPS, and GSM antenna	11.1% FPR and 22% FNR
Mednis et al. [27]	Accelerometer	90% TPR
Bhoraskar et al. [26]	Accelerometer, magnetometer, and GPS	10% FNR
Mohamed et al. [28]	Accelerometer	75.76–87.8% accuracy
Arroyo et al. [13]	Accelerometer, GPS	0.87 AUC, 0.91 recall
Aljaafreh et al. [14]	Accelerometer, smart phone	N/A
Silva et al. [8]	Accelerometer	0.70–80% accuracy
Astarita et al. [16]	Accelerometer, smart phone	90% accuracy, 35% FP
González et al. [17]	Accelerometer, gyro, smart phone	0.82–0.944 AUC
Proposed approach	Accelerometer, gyro, and GPS	97.14% accuracy, FPR < 0.018%, AUC of 0.9784

TPR, FPR, and FNR stand for true positive rate, false positive rate, and false negative rate, respectively.

**Table 5:** Results of the partition bias analysis.

k	Train Data Set	Blind Data Set
1	0.9903	0.9784
2	0.9877	0.9954
3	0.9903	0.9968
4	0.9872	0.9777
5	0.9917	0.9859
Average	0.9894	0.9868

AUC values for the best performance model at each dataset, k *i*th repetition.

**Table 6:** Results of the z-normalization analysis.

k	Train Data Set	Blind Data Set
1	0.9945	0.9731
2	0.9865	0.9806
3	0.9867	0.9761
4	0.9855	0.9845
5	0.9909	0.9924
Average	0.98882	0.98134

AUC values for the best performance model at each dataset, k *i*th repetition, using the Z normalized data sets.
